# Effect of Three Types of Specially-Designed Myopia Control Spectacle Lenses on Astigmatism: A Two-Year Result

**DOI:** 10.1007/s44402-026-00077-5

**Published:** 2026-04-08

**Authors:** Ke Wen, Bi Yang, Longqian Liu, Pauline Cho

**Affiliations:** 1 Department of Ophthalmology, West China Hospital, Sichuan University, Chengdu, China; 2 Laboratory of Optometry and Vision Sciences, West China Hospital, Sichuan University, Chengdu, China; 3 Department of Optometry and Vision Sciences, West China School of Medicine, Sichuan University, Chengdu, China

**Keywords:** Astigmatism, Children, Myopia, Myopia control spectacle lenses

## Abstract

**Purpose:**

This study sought to determine whether specially-designed myopia control spectacle lenses had any effect on astigmatism in clinical practice.

**Methods:**

Medical records of 74 patients (age: 6 to 12 years old) involving the use of MyoVision (*n* = 23), DIMS (*n* = 22) and Stellest lenses (*n* = 29) were collected from the West China Hospital. Data collection included baseline age, sex and spectacle lens prescription at baseline and at the 2-year follow-up. Astigmatism, determined by cycloplegic objective and subjective refraction, was divided into its power vector components, J_0_ and J_45_.

**Results:**

After 2 years of lens wear, for the MyoVision, DIMS and Stellest groups, the total astigmatism increased by −0.41 ± 0.29 D, −0.42 ± 0.34 D and −0.41 ± 0.31 D, respectively (all *p* < 0.001). The J_0_ vector increased by 0.19 ± 0.15 D, 0.19 ± 0.18 D and 0.20 ± 0.16 D, respectively (all *p* < 0.001). The J_45_ vector did not change significantly for MyoVision and Stellest (*p* = 0.12, 0.21), but did decrease significantly with DIMS (*p* = 0.02) although the magnitude (−0.07 ± 0.14 D) was not clinically significant. The changes in total astigmatism, J_0_ and J_45_ were comparable between the three groups (*p* = 0.99, 0.97 and 0.32, respectively). Multivariate linear regression analyses showed that the change in astigmatism was correlated only with the change of SER (standard *β* = 0.31, *p* = 0.006).

**Conclusion:**

The use of specially-designed myopia control spectacle lenses over 2 years resulted in a slight, but clinically insignificant (<0.50 D) increase in refractive astigmatism. The increase was not associated with lens type, but rather with the increase in myopia.

Key Points

- This study investigated changes in astigmatism after wearing myopia control spectacle lenses.
- Statistically, but not clinically significant increases in total astigmatism (<0.50  D) and J_0_ (<0.25 D) were found after 2 years of spectacle lens wear, with negligible changes in J_45_ (<0.10 D).
- The increase in astigmatism was associated with myopia progression, but not lens type.

## Introduction

Myopia is currently a global health challenge, particularly in Asia, with escalating prevalence [1] and earlier onset [2] in children as young as 6 years old [3, 4]. It poses significant risks of high myopia and associated ocular complications later in life [5]. To retard myopia progression, various interventions have been developed that can be categorised into environmental (behavioural), optical, pharmacological and surgical approaches [6]. Among these, an optical intervention, namely specially-designed myopia control spectacle lenses (MCSLs), is increasing in popularity amongst practitioners and patients because of established efficacy and negligible safety risk [7].

MCSL designs employ different mechanisms, including variations of higher-order aberration profiles [8, 9] and modulating retinal contrast [10]. These lenses were first introduced based on evidence that peripheral hyperopic defocus accelerates myopia progression, while peripheral myopic defocus retards myopia progression [11], leading to the development of lenses targeting peripheral myopic defocus. The lenses differ in the ways they generate myopia defocus, including the spatial distribution of the peripheral microstructures and defocus power.

Some large-sample cross-sectional studies have reported no significant differences in astigmatism as a function of age in young children [12, 13]. Further, longitudinal studies suggested that astigmatism remains stable or shows minimal change between 4 and 18 years of age [14, 15]. However, in clinical practice, there are anecdotal reports of children exhibiting a clinically significant increase in astigmatism (≥0.50 D) within 1 year of starting MCSLs wear. Although this does not appear to be a common observation, reports on the effect of MCLSs on astigmatism are scarce, as most reports have focused on changes in spherical equivalent refraction (SER) and axial length. Three studies have reported the effect of MCSLs on astigmatism, including MyoVision (Carl Zeiss, zeiss.com), Stellest (Essilor, essilor.com) and S.T.O.P kit lenses (Zhong Jing Wei Shi (Suzhou), nthopticmed.com) [16–18]; however, their findings were contradictory. Compared to children wearing single-vision spectacles, Xu et al. reported a non-physiological increase in astigmatism [18], whereas Bao et al. and Fedtke et al. did not observe such a change [16, 17]. Hence, it remains unclear whether MCSLs have a significant effect on astigmatism during myopia control treatment. Meanwhile, only Fedtke et al. reported their astigmatic results in terms of vector decomposition. However, this was only a 6-month interim analysis, and therefore, longer-term results are needed. The purpose of the current study is to investigate the effect of the three most common brands of MCSLs used in China on astigmatism (denoted as J_0_ and J_45_ vectors) over a 2-year period, and to determine any association between changes in astigmatism and myopia progression.

## Methods

### Study Design

Data were retrieved from consecutive clinical records of outpatients who commenced use of MCSLs at West China Hospital, Chengdu, between March, 2018 and July, 2021. Inclusion criteria included: (1) age at the first visit: ≤12 years; (2) SER ≤ −0.50 D; (3) monocular best-corrected visual acuity ≤0.00 logMAR and (4) no history of myopia control interventions before using MCSLs. This study was approved by the Ethics Committee of West China Hospital, Sichuan University (Approval No: 2025-1786). Because of the retrospective design, the study was granted a waiver of informed consent by the Ethics Review Committee.

### Data Collection

Pertinent data retrieved included lens type, age at the first visit, sex and cycloplegic spectacle lens prescription at each visit. To measure refractive error, autorefraction (Topcon KR-1; topcon.com) and retinoscopy were performed under cycloplegia using 0.5% tropicamide and 0.5% phenylephrine hydrochloride (Xingqi Pharmaceutical Co. Ltd, sinqi.com). Subjective refinement followed using a trial frame and ophthalmic trial lenses based on the principle of maximum plus to best visual acuity. Only participants without missing data values were included. Participants were categorised into three groups: MyoVision (Carl Zeiss Inc., zeiss.com), Defocus Incorporated Multiple Segments (DIMS) (HOYA Corp., hoyavision.com) and Stellest (Essilor Inc., essilor.com). Details of these lenses are shown in Table 1.

**Table 1:** Characteristics of each lens type.

Lens type	Proprietary peripheral defocus design	Central clear zone	Peripheral zone	Peripheral power
MyoVision(Zeiss)	Asymmetrically distributed progressive defocus	10 mm (horizontal and inferior meridian)	25 mm (horizontal meridian)	Maximum +1.90 D
DIMS (Hoya)	Honeycomb pattern (formed by 400 multiple defocus segments, each 1.03 mm in diameter)	9 mm (in diameter)	33 mm (in diameter)	+3.50 D
Stellest (Essilor)	11 concentric rings (formed by 1021 aspherical lenslets, each 1.10 mm in diameter)	9 mm (in diameter)	57.1 mm (in diameter)	+3.00 ~ +5.00 D

*DIMS* defocus incorporated multiple segments.

### Sample Size Calculation

It was desired to have 90% power for the detection of a clinically significant change in astigmatism of 0.50 D after 2 years, with a referred SD of 0.51 D [16] and a significant two-sided *p*-value of 0.05. A sample size of at least 13 participants per lens group was required.

### Statistical Analysis

Only data from the right eyes were used for statistical analysis. Based on the axis of the negative correcting cylinder, astigmatism was classified as ‘with-the-rule’ (WTR) astigmatism (negative cylinder axis between 0–30° or 150–180°), ‘against-the-rule’ astigmatism (negative cylinder axis between 60 and 120°) and ‘oblique’ astigmatism (negative cylinder axis between 31–59° or 121–149°). Astigmatism was converted into power vector components: J_0_ and J_45_ [19] for analysis. Data were described as mean ± standard deviation (SD) or median (interquartile range, IQR), as appropriate. A chi-squared test was performed to compare sex, as well as changes in the magnitude and axis of astigmatism between groups. Continuous variables among groups were compared using one-way analysis of variance (ANOVA) or Kruskal–Wallis H Test. An independent samples *t*-test or Mann–Whitney U-test was applied for post-hoc comparisons. A paired *t*-test or Wilcoxon signed-rank test was used to analyse changes in refractive status before and after lens wear. Linear regression models assessed factors associated with the 2-year change in astigmatism. Only variables showing statistically significant associations (i.e., *p* < 0.05) in univariate analyses were added to the multivariate regression model. All analyses were performed using IBM SPSS (version 26.0, ibm.com) and graphing with GraphPad Prism (version 9.0, graphpad.com). The level of statistical significance was set at *p* < 0.05.

## Results

Data was retrieved from the files of 74 patients, with 23, 22 and 29 in the MyoVision, DIMS and Stellest groups, respectively (Table 2). No significant differences were found in regard to sex, age, SER, spherical power, astigmatism, J_0_ or J_45_ vectors at baseline across groups (all *p* > 0.10).

**Table 2:** Demographic and baseline biometric data of participants in each lens group.

Characteristic	MyoVision	DIMS	Stellest	*p*
Sex (male: female)	16: 7	9: 13	14: 15	0.13
Age (years)	11.00 (3.00)	9.45 ± 1.47	9.00 (2.00)	0.12
SER (D)	−3.20 ± 1.64	−2.19 (2.06)	−2.13 (2.44)	0.69
Sphere (D)	−2.82 ± 1.46	−2.00 (1.94)	−2.00 (2.00)	0.53
Cylinder (D)	−0.50 (1.50)	−0.50 (0.50)	−0.50 (0.88)	0.88
J_0_ (D)	0.25 (0.75)	0.25 (0.31)	0.32 ± 0.27	0.86
J_45_ (D)	0.00 (0.00)	0.00 (0.03)	0.01 ± 0.14	0.64

Data are shown as mean ± SD or median (interquartile range).

*D* dioptre, *DIMS* defocus incorporated multiple segments, *SER* spherical equivalent refraction.

**Fig. 1:**
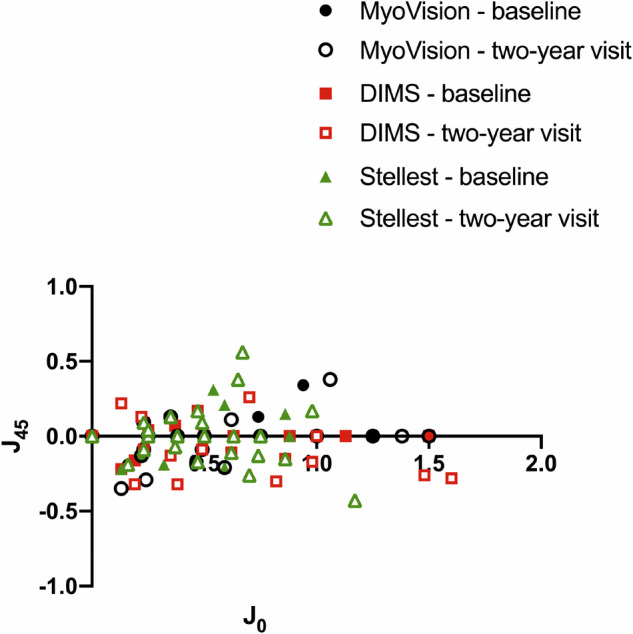
The J_0_ and J_45_ vectors of participants in each lens group at baseline and after 2 years.

**Fig. 2:**
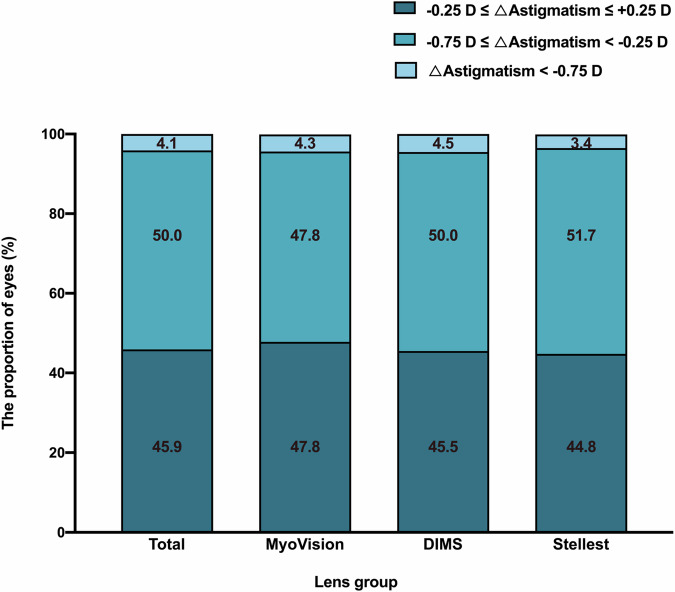
Proportions of change in astigmatism in each lens; △Astigmatism indicates the change in astigmatism after 2 years.

In each lens group, SER and astigmatism (including the J_0_ vector) increased after 2 years of lens wear (all *p* < 0.05). The J_45_ vector decreased in the DIMS group (*p* = 0.02), but remained unchanged in the MyoVision and Stellest groups (*p* = 0.12 and 0.21, respectively) (Fig. 1). Table 3 presents a summary of the findings. The SER change in the MyoVision group (−1.35 ± 0.77 D) was significantly larger than for the Stellest group (−0.38 (0.88) D) (*p* = 0.002) only, while changes in the DIMS and Stellest groups were not significantly different (*p* = 0.11). The magnitude and proportion of change in each component of astigmatism was comparable across the three lens groups (all *p* > 0.30). In total, 95.9% (71/74) of participants had an astigmatic change ≤0.75 D (Fig. 2). Univariate linear analyses of pooled data from the three groups showed that the 2-year change in astigmatism was significantly correlated with baseline SER (standard *β* = −0.27, *p* = 0.02), baseline spherical power (standard *β* = −0.28, *p* = 0.02) and the 2-year SER change (standard *β* = 0.32, *p* = 0.005), but not with baseline age, astigmatism, the 2-year spherical change or lens types (0.09 < all *p* < 0.95). Further, after inputting the three significant variables into the multivariable linear regression analysis, change in astigmatism was only associated with the change in SER (standard *β* = 0.31, *p* = 0.006). All participants had WTR astigmatism. Pooled data showed no significant change in astigmatic axis over time (all *p* > 0.20), and 91.9% participants had a change of axis from baseline ≤10° (Table 4).

**Table 3:** Changes in refractive errors in each lens group.

	MyoVision	DIMS	Stellest	*p*	*p’*
ΔSER (D)	−1.35 ± 0.77	−0.96 ± 0.73	−0.38 (0.88)	0.006	A vs. B: 0.09
					A vs. C: 0.002
					B vs. C: 0.11
ΔAstigmatism (D)	−0.41 ± 0.29	−0.42 ± 0.34	−0.41 ± 0.31	0.99	
ΔJ_0_ (D)	0.19 ± 0.15	0.19 ± 0.18	0.20 ± 0.16	0.97	
ΔJ_45_ (D)	0.00 (0.04)	−0.07 ± 0.14	0.00 (0.10)	0.32	

Data are shown as mean ± SD or median (interquartile range).

*D* dioptre, *DIMS* defocus incorporated multiple segments, *SER* spherical equivalent refraction.

*p* – Probability value for comparison among the three lens type groups derived from One-way ANOVA or Kruskal–Wallis H Test.

*p’* – Probability value for comparison between groups derived from Independent Samples *t* Test or Mann–Whitney U Test.

**Table 4:** Proportions of axis change in each lens group.

Change of axis	Total	MyoVision	DIMS	Stellest	*p*
Stayed within 10° of baseline	91.9% (68/74)	91.3% (21/23)	86.4% (19/22)	96.6% (28/29)	0.42
Stayed within 5° of baseline	81.1% (60/74)	78.3% (18/23)	77.3% (17/22)	86.2% (25/29)	0.66
No change	55.4% (41/74)	65.2% (15/23)	45.5% (10/22)	55.2% (16/29)	0.21

*DIMS* defocus incorporated multiple segments.

## Discussion

Astigmatism is a refractive error that has both magnitude and direction; most individuals have values between −0.75 D and −0.50 D [20, 21]. When it exceeds 1.50 D, spectacles or contact lenses are usually needed to obtain clear vision. It is important to determine whether MCSLs influence this refractive error, and this may be the first study to provide 2-year longitudinal changes in astigmatism with three commonly used MCSLs in clinical practice. The results revealed a minimal increase in total ocular astigmatism and the J_0_ vector in all three MCSL groups. However, for the J_45_ vector, the DIMS group showed a significant decrease, whereas the MyoVision and Stellest groups showed no significant change.

A number of studies have investigated the 2-year myopia control efficacy of MyoVision [22], DIMS [23–25] and Stellest lenses [25, 26] (Table 5). Previous randomised control trials (RCTs), where participants wearing single-vision spectacles served as controls, showed that MyoVision lenses may not retard myopia progression [22], whereas wearing DIMS and Stellest lenses for 2 years could produce a 52% and 55% control effect, respectively [23, 26]. However, previous studies have not compared all three lens types simultaneously, but rather focused on a comparison of just two lenses. Xu et al. found that the 1-year change in SER for participants wearing Stellest lenses was much smaller (−0.31 ± 0.43 D) than for those wearing MyoVision (−0.68 ± 0.43 D) [18]. Three studies compared DIMS and Stellest data [25, 27, 28]. Guo et al. reported a significant difference in the 1-year SER change between DIMS (−0.34 ± 0.04 D) and Stellest (−0.63 ± 0.07 D) [27]. In contrast, both Lembo et al. and Yang et al. reported the SER changes for DIMS and Stellest were equivalent after 1- or 2-years of lens wear [25, 28]. The present study indicated that myopic progression in SER with MyoVision was greater than for DIMS or Stellest. However, significant differences were only observed between MyoVision and Stellest. Future studies should examine other lens types, such as standard MyoCare, MyoCare S (Carl Zeiss, zeiss.com) and Diffusion Optics Technology lenses (SightGlass Vision, sightglassvision.com). Their demonstrated effectiveness has led to an increase in their popularity [29, 30].

**Table 5:** Summary of relevant clinical studies on the myopia control efficacy of each lens type.

Lens type	Study	Study type	Age (years)	Baseline SER (D)	Two-year SER change (D)
MyoVision (Zeiss)	Kanda et al. [22]	RCT	9.58 ± 1.51	−3.18 ± 0.91	−1.43 ± 0.10
	Present study	Retrospective study	Median (IQR): 11.00 (3.00)	−3.20 ± 1.64	−1.35 ± 0.77
DIMS (Hoya)	Lam et al. [23]	RCT	10.19 ± 1.46	−2.93 ± 1.04	−0.38 ± 0.06
	Lembo et al. [25]	Retrospective study	11.2 ± 2.30	−3.40 ± 1.63	−0.50 ± 0.64
	Liu et al. [24]	Retrospective study	11.02 ± 2.53	−2.78 ± 1.74	−0.88 ± 0.62
	Present study	Retrospective study	9.45 ± 1.47	Median (IQR): −2.19 (2.06)	−0.96 ± 0.73
Stellest (Essilor)	Bao et al. [26]	RCT	10.7 ± 0.20	−2.70 ± 0.14 (−4.75 to −0.75)	−0.66 ± 0.09
	Lembo et al. [25]	Retrospective study	11.4 ± 2.38	−3.60 ± 1.81 (-9.25 to -0.05)	−0.63 ± 0.56
	Present study	Retrospective study	Median (IQR): 9.00 (2.00)	Median (IQR): −2.13 (2.44)	Median (IQR): −0.38 (0.88)

*DIMS* defocus incorporated multiple segments, *IQR* interquartile range, *RCT* randomised control trial, *SER* spherical equivalent refraction, *D* dioptre.

In this retrospective study, examining the astigmatic changes after MCSL treatment, statistically, but not clinically significant 2-year increases (<0.50 D) were found for all three lens groups. But it is unclear whether these increases were due to natural growth. Accordingly, there is a need to compare these data with children not undergoing myopia control therapy. Using RCT data, Bao et al. reported a 2-year increase in astigmatism in both a Stellest (−0.51 ± 0.51 D) and a single-vision spectacle group (−0.46 ± 0.41 D). No significant differences were found between the two groups, indicating that the change in astigmatism after wearing myopia control lenses may be a physiological change [16]. As participants in the current study had the same ethnicity, similar baseline age and SER, the present findings were compared with the single-vision group in Bao’s study [16] (historical control) and no significant differences were found for the three lens groups (MyoVision: 0.05 D, DIMS: 0.04 D, Stellest: 0.05 D). Although Xu et al. reported significant differences between participants wearing single-vision lenses (−0.05 ± 0.33 D) and those wearing either MyoVision (−0.09 ± 0.27 D) or Stellest (−0.15 ± 0.33 D) [18], the differences found did not reach clinical significance. It is also important to note that no significant differences in the change in astigmatism were found amongst the three lens groups in the current study, which suggests that the increase in astigmatism is more likely to be caused by physiological growth, rather than being associated with MCSL type.

How does astigmatism increase physiologically? It is generally believed that astigmatism remains stable or shows a minimal decrease during childhood [14, 15], but controversies persist when children are grouped based on different criteria for analysis. In a study of Tohono O’odham Native American children, in general, clinically stable astigmatism was reported. However, the authors noted a −0.02 D/year decrease in astigmatism in participants between 3 and <11 years of age, with a baseline mean hyperopic SER of +0.93 ± 0.03 D. Similarly, a +0.06 D/year increase was seen in children between 11 and <19 years of age, with a baseline mean myopic SER of −0.44 ± 0.06 D [15]. The authors noted that the physiological increases in astigmatism during childhood and adolescence have also been reported in previous studies which only enroled myopic individuals [16, 18, 31]. Thus, it appears that the astigmatic increase is associated with myopic children. For many years, there has been a discussion about the interaction between astigmatism and myopia, with some researchers suggesting that astigmatism develops as a by-product of spherical ametropia (both hyperopia and myopia) [32, 33], while others suggested that the existence of astigmatism occurs in myopia progression [34–36]. There is a positive relationship between the change of SER and the change of astigmatism, as confirmed by Liang et al. [37] and Fan et al. [34]. However, Liang et al. failed to observe any association between axial elongation and astigmatic changes [37]. Gong et al. also used axial elongation to determine myopia progression and reported that participants with longer axial length showed more rapid astigmatic progression [38]. The increase in total ocular astigmatism may be explained by corneal changes, as observations of an increase in both ocular and corneal astigmatism have been reported consistently [18, 39, 40].

In the current study, an increase in J_0_ was observed, presenting as a shift toward increasing WTR astigmatism, but the J_45_ component showed either a clinically insignificant decrease (−0.07 D) or remained unchanged. These results concur with previous results from longitudinal observations in 6- to 14-year-old children [40–43], and are regarded as normal natural changes because of their clinical insignificance.

There are some limitations in the current study. First, subjective rather than objective data were used for analysis, based on autorefraction and retinoscopy. It is standard in our hospital to record the subjective refraction results in the electronic medical records, whereas the objective refraction is not always recorded. Further, seven optometrists were involved in the assessment of subjective refraction, which could have been a source of variation. However, these were all experienced practitioners following the same standard measurement procedures. In addition, all of the participants underwent refraction after complete cycloplegia, which improved comparability between the multiple refraction measurement methods used and may have increased the accuracy of the results [44, 45]. It may have also reduced bias. Second, corneal and internal (non-corneal) astigmatism findings were lacking. These data, if available, may have revealed the detailed mechanism of the astigmatic change observed after wearing myopia control lenses. Unfortunately, these measurements are not obtained routinely. Third, a single-vision control group was absent. As more and more studies support myopia interventions, continuous long-term wear of single-vision lenses can raise ethical concerns in the real-world setting, especially for children 6–12 years of age. This is the stage when rapid myopia progression often occurs [11]. Therefore, data from Bao et al. were used as a historical control [16]. Fourth, due to the small sample size, any group comparisons should be interpreted with caution. However, the conclusion that the increase in astigmatism was independent of lens type can also be supported by the results of the linear analysis. Lastly, the focus on an East Asian population limits the generalisability of the findings. External validation across diverse ethnicities is warranted.

In summary, the present study revealed that different types of MCSLs have varying myopia control effects. Although astigmatism increased to some extent after wearing MCSLs, the magnitude was comparable with physiological changes reported in previous studies, and there were no significant differences between the lens types. Hence, the change in astigmatism may occur with the development of myopia, rather than the type of MCSL.

## Data Availability

Data are available upon request.
